# An Alternative Cause of Encephalopathy: Valerian Root Overdose

**DOI:** 10.7759/cureus.17759

**Published:** 2021-09-06

**Authors:** Corina Freitas, Subrat Khanal, David Landsberg, Viren Kaul

**Affiliations:** 1 Psychiatry, Crouse Health, Syracuse, USA; 2 Psychiatry and Behavioral Sciences, Upstate Medical University, Syracuse, USA; 3 Pulmonary and Critical Care Medicine, Upstate Medical University, Syracuse, USA; 4 Internal Medicine/Critical Care, Crouse Health, Syracuse, USA; 5 Pulmonary and Critical Care Medicine, Crouse Health, Syracuse, USA

**Keywords:** delirium in icu, complementary alternative medicine, autonomic nervous system dysfunction, gaba receptors, hypnotic

## Abstract

The interest in alternative therapeutics use has increased over the past few decades. Valerian, also known as “plant Valium,” is a popular choice as a natural remedy for insomnia or anxiety. In order to ensure patient safety, clinicians need to be knowledgeable about commonly used alternative therapeutic products, their mechanisms of action, and potential pharmacological interactions. We present an unusual case of encephalopathy due to the combination of Valerian root, a plant with putative sedating properties, along with a natural “γ-aminobutyric acid (GABA) supplement.” This case highlights the importance of thoroughly exploring alternative therapies when evaluating encephalopathy as well as the importance of being educated on the commonly used agents.

## Introduction

The World Health Organization reported in 2000 that even though the world prevalence of use of complementary and alternative medicine (CAM) varied significantly among countries and ranged from 9% to 65%, it did notice a trend in the interest rising as natural therapeutics products were more commonly perceived as safer and more organic alternatives [1]. The Centers for Disease Control (CDC) reported in 2015 that the most commonly used CAM in the United States amongst adults were non-vitamin, non-mineral dietary supplements, with a prevalence of 17.7% [2]. The CDC has reported the incidence of short sleep duration in the United States to be around 35.2% [3]. Approximately, 4.5% of individuals diagnosed with insomnia in the United States have used a CAM therapy to treat their condition [4]. Alternative therapeutics products are not well regulated, and dosages and ingredients vary greatly between preparations. A US study on CAM use and disclosure in patients with cancer revealed that herbal supplements were the most commonly used CAM (11.6% respondents), 18% of the respondents did not disclose CAM use to their physician. The reasons for nondisclosure were cited as the physician not asking (57.4%) or participants thinking their physicians did not need to know (47.4%) [5]. To ensure safe and appropriate treatment, clinicians need to inquire and be knowledgeable about commonly used alternative therapeutic products, their mechanisms of action, and potential pharmacological interactions.

Encephalopathy is a common presenting symptom and can result from myriad etiologies. Commonly performed workup can be helpful in identifying a number of these causes, however, toxicity related to ingested substances, especially due to CAMs is often hard to delineate due to the lack of testing available for these substances. The diagnosis is thus dependent on a thorough historical evaluation, often only clarified during an interview with the patient after the improvement of encephalopathy. We present a demonstrative case of encephalopathy induced by a combination of Valerian Root overdose along with a natural "γ-aminobutyric acid (GABA) supplement," taken in an effort to improve sleep.

## Case presentation

A middle-aged woman with a history of bipolar disorder with catatonia (last episode two years prior) prescribed bupropion and carbamazepine and no other medical comorbidities presented with altered mental status after being found by bystanders driving in circles in the parking lot of a church she visited regularly. EMS reported that on their arrival, she was fully awake and responsive yet non-verbal. As such, she was transported to the hospital to evaluate for potential cerebrovascular events. In the emergency department (ED), she was found to be hypertensive, tachycardic, mydriatic, diaphoretic, agitated, and tremulous, symptoms that continued for 24 hours of her admission. Laboratory testing was significant for a blood ethanol level of 15 mg/dL and a carbamazepine level of 3.7 µg/mL (with a therapeutic range of 0.5 −4 µg/mL). A urine drug screen was ordered, however, unfortunately never obtained. The medical team suspected that her presentation represented acute benzodiazepine or alcohol withdrawal and decided to admit her to the intensive care unit (ICU) for close monitoring and supportive management. She was placed on the Glasgow Modified Alcohol Withdrawal Score protocol and required a total of 12 mg of lorazepam for tremors and anxiety within a 48-hour period. Upon arrival in the ICU, the patient was also noted to be exhibiting paranoia in regards to taking medications and accepting nursing interventions, and a psychiatry consult was generated. On psychiatric examination, the patient presented as a young-looking 48-year-old woman. She was sitting up in bed, very still and with slow movements. She had no observable physical deformities or disabilities. She was dressed in clean hospital-supplied clothing and appeared well-groomed. She did not exhibit any abnormal involuntary movements. Her behavior was withdrawn and sullen. Her attitude towards the examining psychiatrist was guarded and suspicious. A friend was present at her bedside, whom she frequently looked at for encouragement. She fixated intensely and gazed at the examiner prior to providing an answer. Her speech was fluent, of normal tone, but hesitant with a significant delay and poverty of content. When asked questions, she frequently questioned the examiner's reasoning for asking the questions. Her range of emotional expression was incongruent with her stated mood of "fine" and was constricted to fear. Her thinking was slowed, circumstantial, and perseverative about wanting to speak only to the psychiatrist from another facility who cared for her two years ago during her last catatonic hospitalization. Her thought content, presented as paranoid and suspicious, was centered around attempting to identify the "real" reasons behind the psychiatrist's presence and questioned the safety of the unit and the ability of strangers to walk in. She did not exhibit any observable responses to internal or unseen external stimuli. Her cognition was grossly conversationally normal, with a fair ability to concentrate. Slowly, as she answered questions and the following tableau emerged: the patient had been having nightmares and poor sleep for over a month. She recounted a history of domestic physical, sexual, and psychological trauma with nightmares and insomnia, treated with carbamazepine and bupropion. Given her desire to not depend on medications any longer, she started weaning her bupropion and her carbamazepine, all while starting to take an over-the-counter Valerian Root supplement at a dose of 1,000 mg daily at bedtime, in addition to another over-the-counter supplement named "GABA supplement." When her nightmares returned and started worsening in frequency and intensity, she began doubling the Valerian Root supplement dose in addition to continuing the GABA supplement at the recommended dose. Two or three days prior to admission, she stopped taking her carbamazepine and bupropion, her anxiety peaked, and she presented feeling "not like herself," "anxious," and "excitable." The following morning, she sought to go to church but was hazy in her recollection of what happened next. She recalled feeling "slower" and "anxious" being in the ambulance and volitionally refusing to answer the EMS' questions. She vehemently denied any alcohol use history, corroborated by collateral. It was determined that the patient's presentation was due to GABA overdose from sedative-hypnotic toxicity using agents with unregulated and therefore unpredictable pharmacodynamics. Alcohol withdrawal treatment was stopped, and her carbamazepine was restarted. By the third day of admission, the patient's sensorium cleared, and her treatment team felt comfortable discharging her home. Upon discharge, she presented with a full, reactive but intense affect, and an anxious mood related to the circumstances leading to this hospitalization.

## Discussion

The term Valerian is derived from the Latin word "valere" which means "to be in good health." Valerian roots, also colloquially known as "plant Valium," are the roots of the *Valeriana officinalis* plant. Valerian root has been used across the globe for its sedative-hypnotic qualities to aid with insomnia or anxiety. It is ingested as tea made from the plant's dried roots or as commercially available over-the-counter preparations (containing either Valerian root alone or in combination with other plants). The typical dose used for insomnia is 300 - 900 mg, taken 30 minutes to one hour before bedtime. Doses higher than 1060 mg daily are associated with toxicity. Valerian products contain a variety of components, including valeric acid, iridoids, alkaloids, furanofuran lignans, and free amino acids such as GABA, tyrosine, arginine, and glutamine [6]. Valerian has demonstrated affinity at various receptors: serotonin (5HT-5a), GABA A and B, α-amino-3-hydroxy-5-methyl- 4-isoxazolepropionic acid (AMPA), N-methyl-D-aspartate (NMDA), and CCK [7,8]. Clinical studies on various Valerian preparations have not demonstrated any relevant interactions with the Cytochrome P 450 enzyme system [9]. Studies have, however, shown that Valerian use leads to potentiation of the effects of phenobarbital, benzodiazepines, and other central depressants [7,8], likely from additive actions at the GABA receptors. Some trials suggest that Valerian has a mild hypnotic action with improved sleep efficiency, especially with long-term use [7,10]. However, the literature is generally of low quality and does not demonstrate consistent benefit when used for insomnia [6,7]. Toxicity or major side effects with valerian are rare [6,7]. Mild symptoms of toxicity include fatigue, abdominal cramps, chest tightness, lightheadedness, hand tremor, and mydriasis, which usually resolve within 24 hours [6,11]. At higher doses, toxicity can present with headaches, acute or delayed hepatotoxicity, cognitive decline, dry mouth, mood alterations including feeling excited or uneasy, strange and vivid dreams, and increased somnolence [9]. Our patient presented with encephalopathy marked by profound somnolence and autonomic instability, likely due to the additive actions of the high doses of Valerian root consumed along with another unidentified GABA formulation. As far as we know, such a significant side-effect, despite even larger doses of Valerian or GABA supplements, has not previously been reported. The encephalopathy resolved after 36 hours of conservative care and close monitoring in the ICU. Since the initially elevated ethanol level was a diagnostic confounder in this case until further investigations were undertaken, it is important to note that the enzymatic reaction-based ethanol testing can produce false positives with values of 3-30 mg/dl. Such a false minute elevation can be due to interference from multiple causes including hemoglobin, lipids, bilirubin, and the use of alcohol-based cleaning solution. This potential interference is important for clinicians to be aware of since it can lead to misdiagnosis. The alcohol level, in this case, was hence a red herring in this situation.

## Conclusions

Alternative medicines containing Valerian Root can lead to encephalopathy along with a constellation of systemic symptoms at higher doses, especially when co-ingested with other sedative-hypnotic drugs. With the use of CAM increasing globally, ingestion of Valerian root as a cause of encephalopathy is the highlight of this case.

This case also reiterates the importance of history and background information when delineating the cause of encephalopathy, especially since at presentation, the patient may not be able to provide this information and assays to test for ingested CAMs are not widely and commonly available. Readdressing potential causes once a patient's mentation clears, exploring ingestions with family members, and having a structured approach when evaluating patients with encephalopathy based on the acuity of onset and evolution are useful strategies to delineate the etiology of encephalopathy. We also discuss how confounding causes of encephalopathy can delay the etiological diagnosis in cases of encephalopathy. Knowing the limitations of diagnostic tests and investigations when attributing causation of encephalopathy is prudent. In our case, the mildly elevated level of ethanol was a false positive related to the limitations of enzymatic reaction-based testing.
